# Relationships Between Basic Psychological Need Satisfaction, Regulations, and Behavioral Engagement in Mathematics

**DOI:** 10.3389/fpsyg.2022.829958

**Published:** 2022-04-12

**Authors:** Anders Hofverberg, Mikael Winberg, Björn Palmberg, Catarina Andersson, Torulf Palm

**Affiliations:** ^1^Department of Science and Mathematics Education, Umeå University, Umeå, Sweden; ^2^Umeå Science Education Research (UmSER), Umeå University, Umeå, Sweden; ^3^Umeå Mathematics Education Research Centre (UMERC), Umeå University, Umeå, Sweden

**Keywords:** engagement, self-determination theory, mathematics, basic psychological need, regulation, structural equation modeling

## Abstract

Behavioral engagement is a key determinant of students’ learning. Hence, knowledge about mechanisms affecting engagement is crucial for educators and stakeholders. Self-determination theory (SDT) offers a framework to understand one of these mechanisms. However, extant studies mostly consider only parts of SDT’s theoretical paths from basic psychological need satisfaction *via* regulations to student engagement. Studies that investigate the full model are rare, especially in mathematics, and results are inconclusive. Moreover, constructs are often merged in ways that may preclude detailed understanding. In this study, we used structural equation modeling to test several hypothesized paths between the individual variables that make up higher-order constructs of need satisfaction, regulations, and behavioral engagement. Satisfaction of the need for competence had a dominating effect on engagement, both directly and *via* identified regulation. Similarly, satisfaction of the need for relatedness predicted identified regulation, that in turn predicted engagement. Satisfaction of the need for autonomy predicted intrinsic regulation as expected but, in contrast to theory, was also positively associated with controlled motivation (external and introjected regulation). Neither intrinsic nor controlled regulation predicted engagement. Theoretical and method-related reasons for this unexpected pattern are discussed, as well as implications for research and teaching.

## Introduction

Engagement, especially behavioral engagement (Hospel et al., 2016), is crucial for students’ learning (Klem and Connell, 2004). Hence, educators and motivation researchers have long been interested in the mechanisms that regulate students’ engagement. Self-determination theory (SDT; Ryan and Deci, 2000) has at its core individuals’ behavior (i.e., engagement) and how it is influenced by situational and personal factors. The influence of situational factors often refers to the extent to which they contribute to students’ perceived sense of autonomy, competence, and relatedness (i.e., the basic psychological needs, Ryan and Deci, 2017b) while personal factors refer to the type of motivational regulations that guide students’ behavior (i.e., external, introjected, identified, and intrinsic). Thus, SDT offers a theoretical link between the teachers’ behavior in the classroom, students’ perceptions of the classroom environment, their motivational regulations, and their engagement in learning (e.g., Furrer et al., 2014). Several studies have investigated links between, for example, the satisfaction of the basic psychological needs for autonomy, competence, and relatedness (henceforth called “need satisfaction”) and engagement (e.g., Reeve et al., 2004; Froiland and Worrell, 2016; Jang et al., 2016a; Olivier et al., 2021), between need satisfaction and students’ motivational regulations (Ng et al., 2012), or between students’ motivational regulations and their engagement in learning (Maulana et al., 2016; Cai and Liem, 2017). However, although SDT is one of the most widespread motivation theories, research on the full chain of events that lead to engagement in school is scarce (Leo et al., 2020), and—to our knowledge—non-existent in the mathematics subject. In general, need satisfaction is assumed to lead to the integration of values and goals (hence promote more autonomous forms of motivational regulations), which in turn are posited to be associated with desired outcomes, for example, higher engagement and school achievement (Ryan and Deci, 2020). Although these assumptions often seem to hold across different contexts, there are indications that the relationships between regulations and outcomes may differ in both strength and direction between educational cultures. For example, the relationship between regulations and outcomes may differ depending on the perceived reasons for learning (Jang, 2008) or the character of the knowledge that is strived for in school (e.g., Taylor et al., 2014)—implying what character of engagement is required for success—which, in turn, is associated with different types of regulations. Hence, the current scarcity of studies in different contexts precludes us from fully understanding the relationships between need satisfaction, regulations, and outcomes, and thus, how to best support students’ engagement and learning. In the present study, we set out to investigate the full chain of events, from perceived need satisfaction, *via* regulations, to students’ engagement in mathematics learning in Grades 4–8 in a Swedish school culture. Hence, providing a full picture of the relationships between needs, regulations, and behavioral engagement is the main contribution of this article. Moreover, as the educational culture in Sweden may differ in several aspects from many of the previous studies (Hofstede et al., 2010; Hofverberg and Winberg, 2020), this study also provides valuable information to future meta-studies on how relationships between needs regulations and engagement (possibly) differ between contexts.

### Engagement

Engagement has since long been viewed as “a robust predictor of student achievement and behavior in school” (Klem and Connell, 2004, p. 262). Engagement refers to the student’s active involvement in a learning activity and is commonly conceptualized as comprising behavioral, emotional, and cognitive engagement (Fredricks et al., 2004). Out of these three types of engagement, Hospel et al. (2016) singled out in their review behavioral engagement as a key construct for academic achievement. Indeed, there is evidence that behavioral engagement is a stronger predictor of achievement than emotional and cognitive engagement (Ladd and Dinella, 2009; Chase et al., 2014; Stefansson et al., 2016). Behavioral engagement has been operationalized in many different ways, for example, as specific behaviors such as students’ choice to participate in learning activities (Finn and Zimmer, 2012), the effort invested in the activity (Hughes et al., 2008), or time on task (Gregory et al., 2014). Other researchers have combined several types of behavior into a global measure, like Hughes et al. (2011) who combined measures of participation, effort, concentration, and persistence into a joint engagement construct. No matter the operationalizations used, studies have shown positive associations between engagement and numerous outcomes. For example, Reeve and Lee (2014) found that early-semester general engagement predicted mid-semester psychological need satisfaction, mastery goal adoption, self-efficacy, and general engagement. Furthermore, changes in general engagement over the first half of the semester predicted end-of-semester psychological need satisfaction, mastery goal adoption, self-efficacy, and achievement. Hence, changes in general engagement seemed to drive changes in students’ motivation over time. Turning to behavioral engagement, Ladd and Dinella (2009) found, in a longitudinal study from kindergarten to Grade 8, that students who were increasingly behaviorally engaged over Grades 1 through 3 showed higher levels of academic growth over Grades 1–8 and higher levels of achievement in Grade 8 than students who were increasingly disengaged over Grades 1–3. Besides having direct effects on achievement, behavioral engagement also seems to mediate the effects of students’ motivational beliefs on achievement. Froiland and Worrell (2016) found indications that behavioral engagement in learning among Grade 9–12 students mediated the effects of the most autonomous form of motivational regulation in SDT, intrinsic regulation, on their grade point averages (GPA). Although Froiland and Worrell’s (2016) model did not include direct effects of intrinsic regulation on GPA, the results provide partial support for the conclusion of Reeve (2012) that engagement fully mediates the effects of students’ motivation on achievement. Hence, there is substantial evidence that students’ engagement affects achievement on the individual level. Moreover, Skinner and Belmont (1993) found that students’ engagement was promoted by teachers’ provision of structure and autonomy support in class, but also that students who were behaviorally disengaged received less motivational support from teachers. Given the substantial impact of students’ behavioral engagement on central aspects of learning and instruction, understanding the mechanisms that influence students’ behavioral engagement is central for educators and educational researchers.

Students’ level of engagement in learning has been shown to be associated with a range of factors, such as school policies and teaching practices (Finn and Zimmer, 2012; Jang et al., 2016b) and the extent to which those practices are perceived as conducive to the fulfillment of students’ goals (Shih, 2008), beliefs (Goldin et al., 2011), or basic psychological needs of competence, relatedness, and autonomy (Jang, 2008; Jang et al., 2010; Ryan and Deci, 2020). Deci and Ryan (2000) argued that knowledge about the relationship between need satisfaction and regulations is essential if we want to understand the motivational processes associated with wellbeing (Ryan and Deci, 2020), cognition (Manganelli et al., 2019), achievement (Taylor et al., 2014), and engagement (Ryan and Deci, 2020). In what follows, we will provide an outline of SDT and review the research on the relations between the different components of SDT and student engagement in learning.

### Self-Determination Theory

#### Overview and Definitions

SDT distinguishes between intrinsic motivation, where the individual engages in a behavior solely because it is enjoyable, and extrinsic motivation, where the behavior aims at fulfilling a goal that is external to the activity itself, for example, engaging in learning to attain a good grade. However, extrinsic motivation comes in different shapes, differing in terms of the degree to which the goal for the behavior has been internalized, and thus to what degree the behavior is perceived as self-determined (Ryan and Connell, 1989; Ryan and Deci, 2020). The least self-determined form of extrinsic motivation is external regulation, where the individual does not have any personal interest in the activity but act solely to achieve external rewards or avoid a threat. Introjected regulation is more self-determined as the behavior emanates from a personal desire to preserve or strengthen the perceived self-worth. However, as this self-worth is contingent on the behavior being approved by others, and therefore, not entirely controllable by the individual, introjected regulation is often categorized under the umbrella-term controlled motivation, together with external regulation. In contrast, in order of increasing self-determination, identified, integrated, and intrinsic regulation are classified as autonomous motivation as the behavior is based on the identification (identified) and internalization (integrated) of the personal value of the behavior, or the enjoyment the behavior brings in itself (intrinsic), which then becomes more self-determined (i.e., autonomous). The division of regulations into controlled and autonomous forms of motivation is common in contemporary research (e.g., Shih, 2008; Cai and Liem, 2017; Ryan and Deci, 2017a; Manganelli et al., 2019; Mouratidis et al., 2021). Results indicate that autonomous motivation generally produces more favorable outcomes than controlled motivation in terms of, for example, grades (Mouratidis et al., 2021), school satisfaction (Li et al., 2018), positive academic emotions (Ketonen et al., 2018), critical thinking (Manganelli et al., 2019), and study effort (Cai and Liem, 2017; Mouratidis et al., 2018).

#### Basic Psychological Needs Satisfaction and Regulations

According to SDT (Ryan and Deci, 2000), people have an innate tendency for autonomous forms of motivation (Ryan and Deci, 2000). However, whether the student will be autonomously motivated in the classroom depends on to what extent the learning situation is perceived to satisfy the basic psychological needs for competence, autonomy, and relatedness (Deci and Ryan, 2000; Olafsen et al., 2018; Ryan and Deci, 2020; Vansteenkiste et al., 2020). It is however unclear whether all basic psychological needs need to be satisfied for students to be autonomously motivated, or if it is sufficient if one or two needs are satisfied. Moreover, results regarding the association between specific needs and regulations are still somewhat inconclusive.

For the sake of brevity, in what follows, we will use the notion “perceived” interchangeably with “the satisfaction of the need for…” Hence, for example, “perceived autonomy” in this paper is identical to a feeling that one’s basic psychological need for autonomy is satisfied. In their review, Ryan and Deci (2000) argued that although perceived competence and relatedness could be sufficient for controlled forms of motivation, perceived autonomy is needed for an internalized regulation. Although several studies have found positive effects of perceived autonomy on identified and intrinsic regulation or autonomous motivation (Ng et al., 2012), most studies also report strong positive effects of perceived competence (Koestner and Losier, 2002; Gnambs and Hanfstingl, 2016; Vasconcellos et al., 2020), while perceived relatedness generally is not essential for autonomous forms of regulations (Koestner and Losier, 2002; Vasconcellos et al., 2020). As to the relative importance of perceived autonomy and competence for autonomous regulations, the results are ambiguous. Intrinsic regulation has sometimes been reported to be substantially more strongly associated with perceived autonomy than with perceived competence (Podlog et al., 2015; Van den Broeck et al., 2016; Kaiser et al., 2020), while equally strong associations with perceived autonomy and competence have also been found (Gnambs and Hanfstingl, 2016).

Identified regulation is sometimes viewed as particularly important in compulsory school, where students might not always be intrinsically motivated. Thus, in these situations, identification and internalization of the goals imposed on the students by the learning environment offers a pathway to autonomous motivation and hence academic achievement. However, there is some ambiguity as to what factors might promote identified regulation. Some studies have shown perceived autonomy to be the strongest predictor of identified regulation by (Podlog et al. (2015), Van den Broeck et al. (2016)), while other studies have found substantially stronger effects by perceived competence (Kaiser et al., 2020).

Turning to the association between basic psychological needs and controlled motivation, perceived autonomy has been found to be negatively related to amotivation and controlled motivation (Ng et al., 2012; Podlog et al., 2015; Van den Broeck et al., 2016). However, some studies have found no effect (Kaiser et al., 2020) or even positive effects (Vasconcellos et al., 2020) of perceived autonomy on external regulation and/or amotivation. Similarly, positive effects of perceived competence on controlled motivation have been found (Ng et al., 2012).

Although results on the relations between needs and regulations are ambiguous, we hypothesize that in our study, perceived autonomy and competence (in that order) will be the main [positive] predictors of intrinsic regulation (H1), in line with most of the studies cited. Perceived autonomy, competence, and relatedness (to a lesser extent than autonomy and competence) will be positive predictors of identified regulation (H2). Further, perceived autonomy will be negatively associated with external and introjected regulation (H3).

#### Regulations and Outcomes

In general, it is assumed that “more autonomous forms of motivation will lead to an enhancement of students’ engagement, learning, and wellness” (Ryan and Deci, 2020, p. 3). Several studies have shown positive relationship between autonomous forms of regulation and outcomes such as academic achievement (Taylor et al., 2014; Froiland and Worrell, 2016), mastery goals (Cai and Liem, 2017), cognitive strategies (Manganelli et al., 2019), and engagement (Shih, 2008; Reeve, 2012; Maulana et al., 2016; Oga-Baldwin et al., 2017; Coelho et al., 2019), while controlled regulations have shown negative relations to these outcomes.

Within SDT research, as in many other theories on motivation, engagement is considered an important outcome of the motivational process. In fact, some researchers have claimed that engagement fully mediates the effect of students’ motivation on achievement (Reeve, 2012). Hence, the relation between regulations and engagement is of particular interest for educators, striving to improve students’ learning. Studies have consistently found a positive relationship between composite measures of autonomous motivation and engagement among students at most stages of the educational system (Jang, 2008; Shih, 2008; Jang et al., 2016a; Cai and Liem, 2017; Coelho et al., 2019) as well as for extracurricular activities such as sport (De Francisco et al., 2020). In Shih (2008), autonomous motivation among Grade 8 students was positively associated with behavioral engagement (involvement, persistence, and participation) and negatively associated with avoidance behaviors. A positive relationship between autonomous motivation and engagement has also been found among students in Grade 4–6 (Cai and Liem, 2017) and children in preschool (Coelho et al., 2019). In terms of individual autonomous regulations, both intrinsic and identified regulation have been shown to be positively associated with engagement (Podlog et al., 2015; Froiland and Worrell, 2016; Maulana et al., 2016; Karimi and Sotoodeh, 2020; Howard et al., 2021).

Controlled motivation in general seems to be negative for engagement, although the picture is somewhat ambiguous. Shih (2008) found that controlled motivation was associated with avoidance behaviors and low involvement, but unrelated to persistence and participation. In Cai and Liem (2017), controlled motivation was negatively associated with engagement only when mediated by self-based goals (e.g., mastery goals). Leo et al. (2020) found no effects of controlled motivation on engagement in in-school physical activities, but positive effects on intentions to participate in out-of-school physical activities. As to the individual constructs of external and introjected regulation, Podlog et al. (2015) found no effects of external regulation on engagement, while Howard et al. (2021), in a meta-study, found weak negative effects of external motivation and moderately positive effects of introjected regulation.

For this study, we hypothesize that controlled motivation either has no or negative effects on engagement (H4), while intrinsic and identified regulation will be positively related to engagement, with intrinsic regulation having a stronger association than identified (H5).

#### Relationships Between Needs, Regulations, and Engagement

##### Direct Effects of Needs on Engagement

Studies based on overall measures of need satisfaction and regulations, respectively, show a relatively consistent pattern of positive associations between need satisfaction and outcomes. For example, in a longitudinal study, Jang et al. (2016a) showed that students’ “overall” need satisfaction was positively associated with a composite measure of behavioral, cognitive, agentic, and emotional engagement. In contrast, studies on the relationship between individual needs and regulations are inconclusive. Several studies have focused on satisfaction of one need at a time, finding positive direct effects on engagement for both relatedness (Martin and Dowson, 2009; Furrer et al., 2014) and perceived autonomy (Núñez and León, 2019). Podlog et al. (2015), who built separate structural equation models to assess the relationships of the respective needs with the individual regulations and with engagement, found more than four times stronger effects on engagement for perceived autonomy and competence than for relatedness.

In contrast, both Raufelder et al. (2014) and Benlahcene et al. (2020), who included all three needs in their models, found significant effects for perceived competence and relatedness on engagement, but not for perceived autonomy. Molinari and Mameli (2018), who excluded autonomy due to poor statistical measurement validity, found that perceived competence was a significant predictor of emotional and behavioral engagement while perceived relatedness was not. Hence, between studies, perceived competence is consistently and positively associated with engagement, while the patterns for autonomy and relatedness are more ambiguous. For our study, we therefore hypothesize that perceived competence will show the strongest positive association with students’ behavioral engagement, followed by perceived relatedness and, lastly, autonomy (H6).

##### The Mediating Role of Regulations

Surprisingly few studies have included the full sequence of relations between psychological need satisfaction, regulations, and outcomes in the education context (Leo et al., 2020). Even fewer studies have examined the relations between the individual psychological needs and regulations as predictors of student engagement in a joint model. Using composite measures of psychological need satisfaction and regulations, Leo et al. (2020) and De Francisco et al. (2020) found that autonomous motivation partially mediated the effects of general need satisfaction on behavioral engagement. Neither Leo et al. (2020) nor De Francisco et al. (2020) found any significant effects of controlled motivation on engagement. Ntoumanis (2005) found that the effect of need satisfaction was almost fully mediated by self-determined motivation (represented by a Relative Autonomy Index-like measure, see Howard et al., 2020).

When measured as individual regulations, intrinsic regulation seems to have a central role in the mediation of effects of need satisfaction on engagement, showing significant mediation effects when considered in isolation (Karimi and Sotoodeh, 2020) and substantially higher mediation effects than other regulations when considered together (Podlog et al., 2015). Karimi and Sotoodeh (2020) found only small indirect effects of perceived autonomy, competence, and relatedness, *via* intrinsic regulation. Unlike most other studies, Podlog et al. (2015) separately measured satisfaction of the basic psychological needs and the regulations (intrinsic, identified, and external regulation, and amotivation). Separate structural equation models were built for each need to assess their respective relationships with regulations and engagement. Intrinsic regulation was found to have a central role in the mediation of the effects of all three needs on engagement. Identified regulation only mediated the effects of relatedness and competence, and to a substantially lower extent than intrinsic regulation did. External regulation had no own significant effect and did not mediate effects of any need on engagement.

Thus, individual needs and regulations seem to differ in their relationships with each other and with engagement. As studies on this topic are scarce and to some extent inconclusive, more studies incorporating the relationships and effects of separate needs and regulations on students’ engagement are warranted. The few existing studies that have included individual needs and regulations indicate that intrinsic regulation and to some extent identified regulation, in addition to their own direct effects on students’ engagement, mediate a substantial part of the effect of need satisfaction on engagement. Controlled motivation and external regulation, in contrast, do not seem to have any substantial own effects but have in few cases been shown to mediate effects of need satisfaction on engagement.

Following the general pattern of the cited studies, we hypothesize that only intrinsic and identified regulation will mediate the effects of psychological need satisfaction on engagement (H7).

### Aim

The aim of this study is to add to our understanding of the associations between students’ need satisfaction, types of motivation regulation, and behavioral engagement in mathematics. Based on the previous research, mostly focused on a small subset of these associations, we constructed the hypotheses listed below.

### Hypotheses

To summarize from previous sections, our hypotheses are as:

*H1*: Perceived autonomy and competence, in that order, will be the main [positive] predictors of intrinsic regulation.

*H2*: Perceived autonomy, competence, and relatedness (to a lesser extent than autonomy and competence) will be positive predictors of identified regulation.

*H3*: Perceived autonomy will be negatively associated with external and introjected regulation.

*H4*: External and introjected regulation either have no or negative effects on engagement.

*H5*: Both intrinsic and identified regulation will be positively related to engagement, but intrinsic regulation will have a stronger effect on engagement than identified regulation.

*H6*: Perceived competence will show the strongest positive association with students’ engagement, followed by perceived relatedness and, lastly, perceived autonomy.

*H7*: Intrinsic and identified regulation will mediate the effects of psychological need satisfaction on engagement.

## Materials and Methods

### Procedure and Participants

Data on students’ need satisfaction, regulations, and behavioral engagement were collected through a web questionnaire from middle of March to late April 2020. The questionnaire was administered to all 3,842 students in municipal schools in Grade 4–8 (*n* = 700–800 in each grade) in a medium sized municipality in the north of Sweden. The students were attending 192 different classes in 30 different schools.

The research project was conducted in accordance with Swedish laws as well as the guidelines and ethics codes from the Swedish Research Council that regulate and place ethical demands on the research process.^1^ For the type of research conducted in this study, it is not necessary to apply for ethical evaluation to the Swedish Ethical Review Authority. Additionally, documented consent from students and legal guardians is not mandatory for this type of questionnaire and sample size, but both students and legal guardians were informed in written form about the study’s aims, possible benefits and risks, and that participation was voluntary. All data were pseudonymized before analyses, so that individuals could not be identified in the results. Before the administration of the questionnaire, each mathematics teacher got instructions to give their students opportunity to respond to the questionnaire during class time. The teachers also received a script for what to say when introducing the questionnaire to students, with information about, for example, how to interpret the answer options, a reminder that participation was voluntary, that they would have no disadvantage if they did not participate, and that the teacher would not see their answers. When applicable, a reminder to let the students complete the questionnaire was sent to teachers 2 weeks after the initial distribution of the questionnaire.

All in all, 1,195 students from 90 classes in 21 schools answered the questionnaire. Out of these, 50 students were classified as careless responders as their responses were classified as outliers in the number of identical answers in a row (i.e., they were outliers in a longest string measure, see Johnson, 2005; Meade and Craig, 2012). Furthermore, 67 students’ answers were considered outliers based on Mahalanobis distance (Meade and Craig, 2012). Students defined as outliers in either longest string or Mahalanobis distance (or in a few cases both measures) were removed from the sample. The sample used in analyses therefore consisted of 1,081 students from 89 classes in 20 schools (*n* = 210 in Grade 4, *n* = 247 in Grade 5, *n* = 270 in Grade 6, *n* = 159 in Grade 7, and *n* = 195 in Grade 8).

### Measures

The questionnaire we used comprised 22 items (Supplementary Table 1). All items were statements that the students were asked to rate to what extent they agreed with on a scale from 1 (not at all) to 5 (fully agree). Satisfaction of students’ need for autonomy, competence, and relatedness was measured with three items for each need. The items used to capture students’ need satisfaction were adaptations from questionnaire items that has previously been used to this end (Williams and Deci, 1996; Deci and Ryan, 2000). Three different types of regulations were also measured as: intrinsic, identified, and controlled regulation. The items measuring regulations were adaptations of a subset of items from the Self-Regulation Questionnaire (Ryan and Connell, 1989). Intrinsic and identified regulations were measured with three items for each construct, while controlled motivation was measured as a composite of two external regulation items and two introjected regulation items. Optimally, external and introjected regulation should be kept separate. However, because of the nested structure of the data, we deemed it necessary to apply analyses that acknowledged clustering (see section “Method of Analysis”). Consequently, we had to limit the number of free parameters in the model compared to the number of clusters to be able to estimate reliable standard errors. As preliminary analyses indicated that external and introjected regulation were highly associated with each other, we decided to combine them into one construct. As introjected and external regulation were hypothesized to follow the same patterns (see hypotheses H3 and H4), evaluating them as a single construct did not change our hypotheses. Behavioral engagement was measured with three items. The items measuring behavioral engagement were Swedish adaptations of a subset of items from the questionnaire of Skinner et al. (2008) on behavioral engagement.

Adaptations of all items were made to suit the context of the participants and the items were tried out in several iterations with a total of 427 students and their teachers in 20 classes. Besides letting their students test preliminary versions of the questionnaire, teachers also helped with assessments of the appropriateness of the items for different grade levels, ensuring that they were both intelligible and easy to read for students in Grade 4–8. Analysis of student data from these pilot tests informed both the selection and formulation of items.

### Method of Analysis

**Figure 1:**
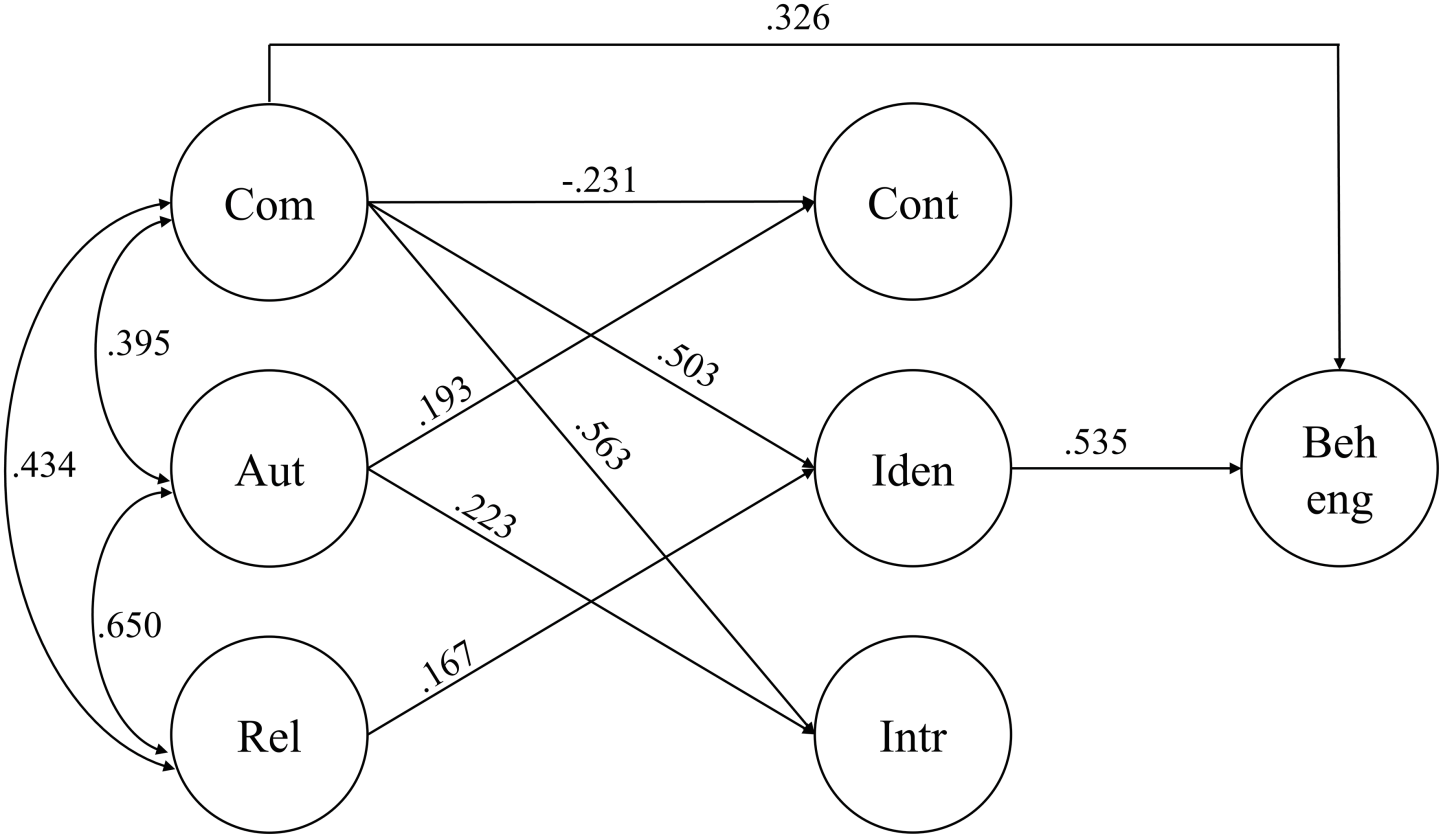
Path diagrams describing the structural relation between the latent variables of the original model (to the left) and the alternative model (to the right). The measurement model, including all observed variables, is excluded in this diagram. Com, perceived competence; Aut, perceived autonomy; Rel, perceived relatedness; Cont, controlled motivation; Iden, identified regulation; Intr, intrinsic regulation; and Beh eng, behavioral engagement.

To examine the associations between students’ need satisfaction, types of motivation, and behavioral engagement, we used structural equation modeling (SEM). We followed a three-step process for these analyses. The first step was to specify a measurement model, where all latent variables were allowed to covary freely with each other. An acceptable fit in this step indicates that the observed variables fit well into the latent constructs and is therefore a prerequisite for a well-fitting model when structural paths are added. The second step was to test the measurement model for measurement invariance (MI) between the different grades. Three levels of measurement invariance were tested: configural, metric, and scalar invariance (Vandenberg and Lance, 2000). If the results support measurement invariance, it shows that the instrument used and the model constructed are invariant in all grades. The third and last step is to add the hypothesized structural relations to the measurement model to make it a complete SEM model. We constructed a model where the three types of need satisfaction covaried with each other. The three types of regulations were regressed on the three types of need satisfaction, and behavioral engagement was regressed on the regulations (see Figure 1, where the structural part of the model is described). An alternative model, identical to the first but with the addition of direct paths from need satisfactions to behavioral engagement, was also constructed. It is these two SEM models, henceforth referred to as the original model and the alternative model, that are used to evaluate our hypotheses.

As is often the case with student samples in educational research, the students in our study were nested in classes and in schools. To examine whether this nesting of students was strong enough to affect the analyses, we calculated two types of intraclass correlations (ICCs) in accordance with Bliese (2000). ICC(1) can be interpreted as the proportion of the total variance that can be explained by group membership. ICC(2) can be interpreted as the reliability of the group-level means. The literature does not provide clear guidelines on how high ICC values should be to indicate that the researchers need to take nesting into account in analyses, but Lam et al. (2015) suggested that ICC(1) exceeding 0.1 and ICC(2) exceeding 0.7 show sufficient group-level variability and reliability to use multilevel analyses.

A drawback of using ICCs is that the bias that cluster size contributes with is ignored. Therefore, we also calculated the design effect (deff). The design effect is a measure of the sample’s deviation from a simple random sample (Lai and Kwok, 2015). As for ICCs, there are no clear cut-off for design effect. There is a popular rule of thumb that single level analyses do not lead to misleading results when deff is below two. Lai and Kwok (2015) test this rule of thumb and found that it was applicable under several conditions, although they recommended that single level analyses are used only when deff values are lower than 1.1.

Analyzing the nesting of students in classes, we found that ICC(1) values ranged between 0.04 and 0.22, ICC(2) values between 0.31 and 0.78, and deff values between 1.39 and 3.48. Consequently, the nested structure of the data could not be ignored. However, since group-level associations were outside the aim of this study, and since multilevel analyses may be biased when group sizes are as small as in our case (under 30 individuals per cluster) and ICC(1) values as low as ours (0.2 or lower; Hox and Maas, 2001; Maas and Hox, 2004), we did not use multilevel modeling. Instead, we used the “complex” option in Mplus 8.4, which adjusts standard errors for the bias arising on individual level because of the nested structure (McNeish et al., 2017) and performs well as long as there are 25 clusters or more (Huang, 2018). The complex option compensates standard errors for effects from the nesting, but estimates are not affected. We also used the robust maximum likelihood estimator as it is robust against violations of normality and can handle missing data through the full information maximum likelihood method. The amount of missing data ranged between 0.2% and 5.2% on individual items.

Model fit was evaluated through chi-square values and four goodness-of-fit indices: root mean square error of approximation (RMSEA), standardized root mean square residual (SRMR), comparative fit index (CFI), and Tucker-Lewis index (TLI). Acceptable values for both RMSEA and SRMR are <0.08 (Browne and Cudeck, 1992; Hu and Bentler, 1998), even if RMSEA <0.06 is more desirable (Hu and Bentler, 1999). For CFI and TLI, the cut-off >0.90 is often used, as models with values <0.90 generally can be significantly improved (see Bentler and Bonett, 1980), but >0.95 would be more desirable (Hu and Bentler, 1999). When evaluating whether the data supported measurement invariance over the grades, we also considered the deterioration of fit between measurement invariance models (i.e., between the configural, metric, and scalar invariance model; Vandenberg and Lance, 2000). The change in CFI (ΔCFI) should be smaller than or equal to −0.01 to support invariance (Cheung and Rensvold, 2002).

When we compared the original model (see Figure 1) and the alternative, more complex model we compared the fit indices mentioned above but also the Akaike information criterion (AIC) and Bayesian information criteria (BIC). Moreover, we conducted a chi-square difference test (Satorra and Bentler, 2010) to test whether the difference in fit was significant.

## Results

Descriptive statistics (means, standard deviations, and zero-order correlations) for the constructs used in analyses are presented in the Table 1.

**Table 1:** Pearson correlations and descriptive statistics for basic psychological needs satisfaction, types of regulations, and behavioral engagement.

	Variable	1	2	3	4	5	6	7	*M*	Range	SD
1	Competence	-	0.327^**^	0.366^**^	−0.150^**^	0.480^**^	0.539^**^	0.564^**^	3.92	1–5	0.88
2	Autonomy		-	0.545^**^	0.038	0.282^**^	0.362^**^	0.317^**^	2.52	1–5	0.92
3	Relatedness			-	−0.043	0.364^**^	0.303^**^	0.357^**^	3.81	1–5	1.03
4	Controlled				-	−0.189^**^	−0.079^*^	−0.122^**^	2.37	1–5	0.98
5	Identified					-	0.493^**^	0.666^**^	4.07	1–5	0.92
6	Intrinsic						-	0.503^**^	2.64	1–5	1.24
7	Behavioral engagement							-	3.74	1–5	0.84

*The correlation analysis is based on the mean of the individual items of each latent variable. M, mean and SD, standard deviation*.

* *p < 0.01*;

** *p < 0.001*.

### Measurement Model and Measurement Invariance

The data fitted well with the measurement model, where all latent variables were allowed to covary freely (see Table 2). The chi-square value was significant, indicating a lack of fit, but this is expected from anything but perfect models when sample sizes are relatively large (Bentler and Bonett, 1980). Furthermore, all items had substantial loadings on their designated latent variable (all standardized loadings were within the span 0.56–0.94, see Supplementary Table 2). Thus, we deemed it justified to move on to the second step: MI testing. The results of the MI testing are presented in Table 2 and show that the data supported configural, metric, and scalar invariance between Grades 4 and 8. We therefore proceeded with the full SEM analyses.

**Table 2:** Fit statistics for the measurement model and measurement invariance models across grades.

Model	*k*	*χ* ^2^			RMSEA		CFI	TLI	SRMR	ΔCFI^a^
		Value	*df*	*p*	Value	90% C.I.				
Measurement model	87	545.1	188	<0.001	0.042	0.038–0.046	0.972	0.965	0.057	-
Measurement invariance across grades										
Configural	435	1,466.4	940	<0.001	0.051	0.046–0.056	0.956	0.946	0.069	-
Metric	375	1,570.4	1,000	<0.001	0.051	0.046–0.056	0.952	0.945	0.076	−0.004
Scalar	315	1,679.7	1,060	<0.001	0.052	0.047–0.057	0.948	0.943	0.078	−0.004

*k, number of parameters; df, degrees of freedom; RMSEA, root mean square error of approximation; C.I., confidence interval; CFI, comparative fit index; TLI, Tucker–Lewis index; and SRMR, standardized root mean square residual.*

a *Only applicable on the measurement invariance models. ΔCFI describes the difference in CFI from the less restrictive measurement invariance model (*i.e.*, the metric model compared with the configural, and the scalar model compared with the metric)*.

### Comparing Models With and Without Direct Effects

**Figure 2:**
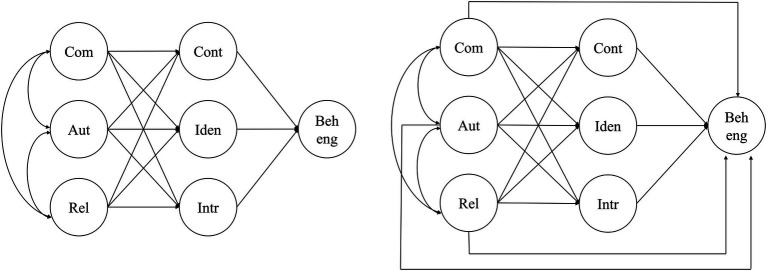
Path diagram describing the structural relations between the latent variables of the alternative model. All observed variables are excluded in this diagram, and only significant paths are shown (*p* < 0.05). All coefficients are standardized. Com, perceived competence; Aut, perceived autonomy; Rel, perceived relatedness; Cont, controlled motivation; Iden, identified regulation; Intr, intrinsic regulation; and Beh eng, behavioral engagement.

Both the original SEM model and the alternative, more complex, model that included direct effects from need satisfaction on engagement fitted the data well (see Table 3). Like in the measurement model, the standardized loadings of the observed variables on the latent variables were substantial (i.e., between 0.57 and 0.94, see Supplementary Table 2). Comparing the two models, the alternative model had a slightly better fit than the original. A chi-square difference test also confirmed that the chi-square value was significantly better for the alternative model, *χ*^2^(3) = 42.67, *p* < 0.001. Moreover, there was a significant direct path between perceived competence and behavioral engagement in the alternative model (see Figure 2). We concluded that the more complex model described the associations between need satisfaction, regulation, and behavioral engagement better than the original model did. This also shows that students’ regulations do not fully mediate the effect of need satisfaction on behavioral engagement, although the direct effect of both relatedness and autonomy on engagement was insignificant.

**Table 3:** Fit statistics for the original model and the alternative model.

Model	*χ* ^2^			RMSEA		CFI	TLI	SRMR	AIC	BIC
	Value	*df*	*p*	Value	90% C.I.					
Original model	680.7	194	<0.001	0.048	0.044–0.052	0.961	0.954	0.066	58690.0	59093.8
Alternative model	616.0	191	<0.001	0.045	0.041–0.049	0.966	0.959	0.065	58618.7	59037.5

*Original model, only paths between needs and regulations, and between regulations and engagement; Alternative model, the original model with the addition of direct paths from basic psychological needs satisfaction to behavioral engagement; df, degrees of freedom; RMSEA, root mean square error of approximation; C.I., confidence interval; CFI, comparative fit index; TLI, Tucker–Lewis index; SRMR standardized root mean square residual; AIC, Akaike information criterion; and BIC, Bayesian information criterion*.

### Basic Psychological Need Satisfaction and Motivational Regulations

The final model, including direct paths from basic psychological needs to behavioral engagement, is presented in a simplified version (non-significant paths excluded, for the sake of parsimony) in Figure 2. Generally, our model explained the variance in identified (*R*^2^ = 0.37) and intrinsic (*R*^2^ = 0.43) regulation better than the variance in controlled motivation (*R*^2^ = 0.06). Of the three basic psychological needs, perceived competence was a more important predictor of students’ regulations and behavioral engagement than either perceived autonomy or relatedness. Perceived competence was significantly associated with all outcomes, positively with behavioral engagement, identified regulation, and intrinsic regulation but negatively with controlled motivation. The only significant path from perceived relatedness was a weak positive association with identified regulation, and perceived autonomy was positively associated with intrinsic and controlled regulation. Although perceived autonomy was significant for intrinsic motivation and perceived relatedness was significant for identified motivation, the path coefficients from perceived competence were substantially higher in both cases.

### Motivational Regulations and Behavioral Engagement

In total, our model explained a large proportion of the variance in behavioral engagement (*R*^2^ = 0.69), although Figure 2 shows that identified regulation was the only regulation to have a significant effect on behavioral engagement (*β* = 0.535, *p* < 0.001).

### Basic Psychological Need Satisfaction and Engagement

As mentioned under section “Basic Psychological Need Satisfaction and Motivational Regulations”, perceived competence was the only basic psychological need with significant direct effect on behavioral engagement. When considering both indirect and direct effects on engagement (see Supplementary Table 3 for a list of all coefficients), competence had a combined standardized path coefficient of 0.626 (indirect effect = 0.300, direct effect = 0.326), autonomy 0.080 (indirect effect = 0.038, direct effect = 0.042), and relatedness 0.094 (indirect effect = 0.084, direct effect = 0.010). This strengthens the fact that competence need satisfaction was the most important basic psychological need for students’ behavioral engagement.

Perceived relatedness was more important for students’ behavioral engagement than the need for autonomy. Although relatedness did not have a significant direct effect on engagement, the total indirect effect, mediated through the three types of regulations, was significant (*β* = 0.084, *p* = 0.013). A closer inspection reveals that it was the path from perceived relatedness *via* identified regulation to behavioral engagement that contained this positive effect (*β* = 0.090, *p* = 0.005). In contrast, none of the indirect pathways including perceived autonomy were significant.

The indirect effects of need satisfaction on behavioral engagement covered a large proportion of their total effects (48% for autonomous and perceived competence; 89% for perceived relatedness), implying that the effect of need satisfaction was mediated through the regulations. To conclude that there is mediation of an effect, there should be significant paths between both the predictor (need satisfaction) and the mediator (regulations) and between the mediator and the outcome (behavioral engagement). As there were significant paths between perceived competence and identified regulation, between perceived relatedness and identified regulation, and between identified regulation and behavioral engagement, the significance of the total indirect effect was tested. Both the two possible indirect effects (from either perceived competence or relatedness, *via* identified motivation to behavioral engagement) were significant (for competence: *β* = 0.269, *p* < 0.001; for relatedness: *β* = 0.090, *p* = 0.005), indicating mediation.

## Discussion

### Basic Psychological Needs and Regulations (H1–H3)

Concerning the relation between need satisfaction and types of regulation, we hypothesized that perceived autonomy would be a prerequisite for the two types of autonomous motivation (identified and intrinsic regulation). To be more specific, we first hypothesized that both perceived competence and autonomy would be important for intrinsic regulation, perceived autonomy being a stronger predictor than perceived competence (hypothesis H1). Our results partly supported H1, as competence and autonomy were significantly positively related to intrinsic regulation, while relatedness was not. However, contrary to what is stated in H1, perceived competence was a substantially stronger predictor of intrinsic regulation (*β* = 0.563) than perceived autonomy was (*β* = 0.223). Although this result goes against our hypothesis, a recently published meta-analysis by Bureau et al. (2022), that was not published when we formulated our hypotheses, shows that our result is in line with the meta-analytic associations between needs and regulations.^2^

The hypothesis concerning identified regulation (H2) was also partly supported. Again, perceived competence turned out to be the strongest predictor, but perceived autonomy was not significantly associated with identified regulation. Moreover, perceived relatedness was significantly associated with identified regulation, although the association was substantially weaker than for perceived competence. This contradicts the theoretical arguments that the need for autonomy must be satisfied for students to be autonomously motivated (Koestner and Losier, 2002; Ryan and Deci, 2020) but corroborates the results of a recent study by Kaiser et al. (2020). It also resembles the low path coefficient between perceived autonomy and identified regulation found by Bureau et al. (2022). Bureau et al. argued that the salience of achievement in school environments makes the need for competence the driving factor in explaining motivational regulation, even if studies in other contexts (e.g., work, see Van den Broeck et al., 2016) still emphasize the importance of perceived autonomy. The fact that perceived relatedness was a stronger predictor of identified regulation than perceived autonomy was indicates that feeling a connection with the teacher is more important for students’ development of identified regulation than a feeling of autonomy. Intuitively, this seems logical. If students feel a connection with the teacher, they should be prone to identify with the teachers’ values (e.g., the value of learning mathematics) and integrate these values with their own. Hence, our result emphasizes the importance of social aspects of teaching together with the importance of making students feel confident in their own competence.

The third hypothesis concerning the relation between need satisfaction and regulations (H3) stated that perceived autonomy should be negatively associated with external and introjected regulation (in our case, controlled motivation). This hypothesis was not supported by the results, as perceived autonomy instead was positively associated with controlled motivation. Perceived competence, in contrast, was negatively associated with controlled motivation. It is worth mentioning that the explained variance in controlled motivation was low. Still, the positive association between perceived autonomy and controlled motivation may be a product of the relation between perceived autonomy and the introjected component of controlled motivation. Introjection involves a partial internalization of values (Ryan and Deci, 2017a) and may therefore be reinforced by feelings of autonomy. Indeed, there are meta-analytic studies that have found stronger associations between perceived autonomy and introjection than between perceived autonomy and any other form of regulation (Vasconcellos et al., 2020), although these results were within physical education. If this association is significant for introjected regulation, but not for external regulation, it is a reason to separate these two forms of controlled regulations in analyses instead of combining them as we have done. Future studies should examine the unique contributions of different forms of controlled regulations further.

One possible reason that perceived autonomy was not significantly associated with identified regulation, but to the less internalized forms of regulation, is statistical interference due to collinearity between predictors. Although perceived autonomy on its own might be associated with identified regulation, its predictive ability could overlap with that of perceived competence and/or relatedness (i.e., they explain the same portion of the variance in the dependent variable). In the presence of such collinearity, the association between perceived autonomy and identified regulation could be canceled out. Mplus cannot calculate collinearity statistics (e.g., variance inflation factor, VIF) in a SEM model, but an indication of collinearity is the relatively strong zero-order correlation between autonomy and relatedness, and the positive correlation they both have with identified regulation (see Table 1). Further, an *ad-hoc* orthogonal projection to latent structure (OPLS; Trygg and Wold, 2002) analysis (not shown) indicated that perceived autonomy indeed overlapped substantially with perceived relatedness in the prediction of identified regulation, with relatedness being a marginally stronger predictor. These results support our hypothesis that the lack of “effect” of perceived autonomy on identified regulation in the SEM model may be due to statistical interference from perceived relatedness. In contrast, in our SEM model, perceived competence and relatedness had overall weak relations with controlled motivation and therefore interfered to a low extent with the relation between perceived autonomy and controlled motivation. Hence, the fact that perceived autonomy was significantly associated with controlled motivation, but not with identified regulation, could be a result of less interference with other needs rather than an indication of a stronger association with controlled regulation. Both the zero-order correlations (Table 1) and additional SEM analyses, excluding perceived competence and relatedness from the model, confirmed that perceived autonomy indeed was substantially more strongly associated with identified regulation than with controlled motivation, thus corroborating the proposed interference between collinear variables in the SEM model.

### Regulations and Engagement (H4 and H5)

We formulated two hypotheses concerning the association between types of regulations and engagement. Based on previous studies, we hypothesized that external and introjected regulation (i.e., controlled motivation, in our case) either would have no effect or negative effect on engagement (H4). The former proved to be true in our results. Hence, feeling pressured and controlled to do math work did not affect engagement. However, neither did doing math work for intrinsic reasons (i.e., because it is fun and interesting), which is contrary to the second hypothesis (H5). The only type of regulation that significantly predicted behavioral engagement was identified regulation. This is puzzling, since, according to SDT, intrinsic regulation would lead to spontaneous engagement in mathematics learning and several studies cited in our background have shown a positive relation between intrinsic regulation and engagement. Again, an *ad-hoc* OPLS analysis indicated substantial overlap between intrinsic regulation and perceived competence in the prediction of engagement, with competence being the marginally stronger predictor. This make sense as feeling competent is a positive emotion (e.g., flow; Csikszentmihályi and LeFevre, 1989), which are central components of intrinsic regulation. Hence, a sense of satisfaction or joy of working with mathematics could be the shared underlying affective component of perceived competence and intrinsic regulation that is associated with engagement, although only competence gained significance in the prediction due to interference between overlapping variables. Still, even if statistical interference weakened the association between intrinsic regulation and engagement, zero-order correlations (see Table 1) indicated that the association between identified regulation and engagement was stronger than that between intrinsic regulation and engagement. A possible explanation was offered by Losier and Koestner (1999), who found that identified, but not intrinsic regulation, was significantly positively associated with perceived relevance of voting and actual voting behavior, which is a form of engagement. The authors proposed that identified regulation “…is key to the successful regulation of behaviors that are socially valued but not necessarily fun…” (p. 294). This conclusion was later shared by Ryan and Deci (2017a), who stated that in activities that are not inherently fun, identified regulation may be preferable to intrinsic motivation. Our results corroborate this proposition, as identification and internalization of the value of math work, which may not be inherently fun for everyone, seemed to be key for behavioral engagement during mathematics lessons.

As argued by Losier and Koestner (1999) as well as Ryan and Deci (2017a), the nature of the task in which the individual engages may be an important factor in the motivational process. Arguably, the downhill skiing that was in focus in the study of Podlog et al. (2015) could be considered a potentially inherently enjoyable activity and thus explain the strong association between intrinsic regulation and engagement that they found. However, enthusiasm was also part of their engagement measure, which may have further contributed to a strengthened association between intrinsic regulation and engagement in their study. More similar to our results, Howard et al.’s (2021) meta-analysis in a general education-related sample of studies showed that identified regulation was slightly better at predicting engagement than intrinsic regulation was. Hence, we argue the character of the activity remains a plausible moderator of the relative importance of different types of autonomous regulation for engagement in the activity.

### Basic Psychological Needs and Behavioral Engagement (H6)

For the effect of need satisfaction on behavioral engagement, we hypothesized that perceived competence would be more strongly associated with behavioral engagement than perceived relatedness and autonomy would be, and that relatedness would be more strongly related with behavioral engagement than autonomy (H6). Considering only direct effects, this hypothesis was partly supported. On one hand, competence had a direct, positive effect on behavioral engagement, even when the indirect effect through different types of regulation was controlled for. On the other hand, neither autonomy nor relatedness had any significant direct effect on behavioral engagement. Considering both indirect and direct effects, there is no doubt that competence was the most important basic psychological need for predicting behavioral engagement. Relatedness had a slightly stronger combined path coefficient (*β* = 0.094) than autonomy had (*β* = 0.080) and the indirect effect of relatedness on engagement, *via* identified regulation, was significant, so there is some support for that part of the hypothesis too. Still, the difference between relatedness and perceived autonomy was too small to lend full support for the hypothesis. Although the results differ somewhat from the hypothesis, similar results have been reported by Molinari and Mameli (2018) who found that perceived competence was a significant predictor of engagement, but relatedness was not. As discussed above, there was a considerable overlap between perceived competence and intrinsic motivation in their prediction of engagement. Hence, it seems that it was mainly the affective dimension of feeling competent (similar to being intrinsically motivated) that fueled engagement in this study.

### Psychological Needs, Regulations, and Engagement (H7)

Few studies have included the full sequence of relations between psychological need satisfaction, regulations, and outcomes, and even fewer has done this while separating different needs and regulations from each other. Therefore, we only proposed a tentative hypothesis for this whole chain of effects, stating that the two forms of autonomous regulation (identified and intrinsic regulation) would mediate the effect of psychological need satisfaction on behavioral engagement (H7). We conclude from the results that identified regulation had a key role, mediating the effects of both perceived competence and perceived relatedness on behavioral engagement. In contrast, intrinsic regulation did not mediate any effects on behavioral engagement. Hence, hypothesis H7 was partly supported. Thus, although the picture may be more complex than implied by our model (for reasons discussed earlier), it seems that perceived competence, relatedness, and identified regulation are central in students’ engagement in mathematics learning. This result is consistent with previous studies that repeatedly have found perceived competence to be important for student engagement. However, when individual basic needs and regulations have been studied together, intrinsic regulation have been shown to be the major predictor and mediator of the effects of basic needs on students’ engagement (Podlog et al., 2015). As previously discussed, the object of students’ engagement in the study of Podlog et al. was possibly more enjoyable than in our study, which may explain the prominent role of intrinsic regulation in their study. Nevertheless, the role of identified regulation as a mediator of the effects of competence and relatedness on engagement was also present in the results of Podlog et al. Hence, we argue the character of the activity in which students engage seems to moderate the relative importance of identified and intrinsic regulation as predictors and mediators of the effects of basic needs on engagement.

### Limitations

One limitation of all cross-sectional research designs is that causal relations cannot be inferred from the data. Hence, what we sometimes call an effect in our results is an effect only in a statistical sense but do not necessarily correspond to a one-way effect from one variable to another in practice. Thus, there is a need for longitudinal or experimental studies to verify the directionality of these relations.

Another limitation is inherent to the method of analysis chosen for this study. In several cases, variables seemed to overlap each other in the prediction of the dependent variables, and several of the hypothesized relationships may have been hidden by this overlap. Although there are both empirical and theoretical reasons to use individual constructs of, for example, regulations and basic psychological needs, collinearity may be an issue. For example, in our case, the insignificant path between intrinsic regulation and behavioral engagement seems to be the result of an overlap between intrinsic and identified regulation, not a lack of association between intrinsic regulation and behavioral engagement. Similar reasoning applies to the basic needs, where the relationship of perceived autonomy with identified regulation seems to have been masked by perceived relatedness. Hence, caution is warranted when using SEM for analyzing relationships between collinear variables.

### Implications

The hypotheses formulated for this study were based on current knowledge about the relations studied here, but the literature cited in the background of this article was seldom unanimous in terms of the nature of these relations. Despite basing our hypotheses on the current knowledge, we found no support for several of the hypotheses and only partial support for several more. This indicates that the relations between basic psychological needs, types of regulations, and behavioral engagement are still poorly understood and that there are mechanisms that need further investigation. For example, future studies should examine under what circumstances intrinsic regulation is predictive of engagement and mediates the effect of basic psychological needs and when it does not. The seemingly weak role of perceived autonomy in our study is another issue that deserves a closer look as it goes against the basic assumptions of SDT but is repeated in other studies (e.g., Kaiser et al., 2020). Previous results (e.g., Taylor et al., 2014; Kaiser et al., 2020) imply that culture, educational context, age group, study design, and the operationalizations and definitions of constructs may affect results.

One implication of our results is that it is better to separate identified regulation from intrinsic regulation than to aggregate the two into a composite autonomous motivation construct (see also Howard et al., 2017, 2020). The two types of autonomous motivation had unique antecedents and outcomes. Therefore, combining the two leads to a loss of important information and possibly erroneous conclusions. For example, both Shih (2008) and Coelho et al. (2019) concluded that autonomous motivation predicts engagement, while Wilson et al. (2012) concluded that autonomous motivation did not mediate the effect of transformational teaching on student engagement. It is possible that only one of the regulations included in the autonomous motivation measures used in these studies predicted engagement or mediated the effect on engagement, and therefore, these conclusions could have been different if intrinsic regulation was separated from identified regulation. Although we acknowledge that combining constructs may be necessary for measurement reasons, just like we combined external and introjected regulation in our study, we encourage researchers to keep the regulations separate when possible.

It is important to note that although perceived autonomy and intrinsic regulation were of seemingly little importance in this study, there is plenty of support for their importance in students’ academic and personal development and wellbeing. Therefore, our results should not be interpreted as diminishing the importance of providing autonomy support and fostering students’ intrinsic regulation. Also, because of statistical interference between predictors, perceived autonomy may have stronger positive relations with identified regulation, and thus with engagement, than indicated by the SEM model. Similarly, intrinsic regulation may have stronger relation with engagement than indicated. Thus, for example, if teachers could make students feel intrinsically regulated to a higher extent, it could have a positive effect on their engagement. The same applies to students’ identified regulation. However, if both are “turned up” at the same time, the effect will not be additive as their effects would overlap.

For teachers, our results imply that to get students engaged in the math lessons, it is crucial to provide students with feedback that helps them develop a sense of competence in doing mathematics. Our study is cross-sectional and does not warrant conclusions about causality, but the theoretical—with varying strength, empirically supported—predictions of causal relations which our models are based on do account for the variation in our data in a satisfactory way. If these causal relations hold, feelings of competence will not only strengthen autonomous forms of motivational regulations, but it will also have a direct effect on students’ engagement in the classroom. To a lesser degree, it is also important to show the students that you care about them and make them feel appreciated as this will help them identify with and internalize the goals of the teaching. Identified regulation was an important predictor and could therefore be targeted, but according to our results, supporting students’ need for competence still seems to be key for students’ behavioral engagement as it also seems to promote identified regulation.

## Data Availability Statement

The pseudonymized raw data supporting the conclusions of this article will be made available by the authors upon request, without undue reservation.

## Ethics Statement

Ethical review and approval was not required for the study on human participants in accordance with the local legislation and institutional requirements. Written informed consent from the participants’ legal guardian/next of kin was not required to participate in this study in accordance with the national legislation and the institutional requirements.

## Author Contributions

TP, BP, CA, and MW contributed to the conception and design of the main study of which the present study is a part. BP, TP, and MW designed the instruments and collected the data. TP, BP, MW, and AH drafted the overarching research questions for the present study. AH had the main responsibility for designing the methodology, creating models, and performing the analyses reported in this manuscript and wrote the first draft of the manuscript. MW wrote large parts of the introduction and background. BP and TP wrote certain sections in the background and method. There was close collaboration between the authors throughout the process and all authors took part in discussing the results, manuscript revision, and reading. TP acquired the funding and managed the main project leading to this publication. All authors contributed to the article and approved the submitted version.

## Funding

This study was funded by the Swedish Research Council under grant number 2019-04349. Funding for open access publication was supplied by Umeå University.

## Conflict of Interest

The authors declare that the research was conducted in the absence of any commercial or financial relationships that could be construed as a potential conflict of interest.

## Publisher’s Note

All claims expressed in this article are solely those of the authors and do not necessarily represent those of their affiliated organizations, or those of the publisher, the editors and the reviewers. Any product that may be evaluated in this article, or claim that may be made by its manufacturer, is not guaranteed or endorsed by the publisher.

## Footnotes

^1^ http://www.codex.vr.se/en/

^2^We would like to thank the reviewer that suggested this newly published article to us.
