# Supplementary material for: Relationships Between Basic Psychological Need Satisfaction, Regulations, and Behavioral Engagement in Mathematics
Source: Front Psychol. 2022 Apr 12;13:829958. doi: 10.3389/fpsyg.2022.829958 (PMC9040704; doi:10.3389/fpsyg.2022.829958)
Supplement: Supplementary file 1 [file Table_1.pdf]

Supplementary Table 1. Items Used to Assess Basic Psychological Needs, Regulations, and Behavioral Engagement

Construct	Label	Full item (translated into English)
Basic Psychological Need		
Competence	c2	I feel that I can perform well in mathematics.
Competence	c3	I believe I can become as good as I want in mathematics.
Competence	c4	I am sure I will be able to understand what we work with in the math lessons.
Relatedness	r1	I feel that my teacher cares about me.
Relatedness	r2	My teacher makes an effort to make me feel good.
Relatedness	r3	My teacher thinks I am important.
Autonomy	a2	If I would tell my teacher what I would like to do on the next math lesson, there is a big chance we will do that.
Autonomy	a3	My teacher would think that it was good if I suggested what we could do on the math lessons.
Autonomy	a4	I am allowed to participate in decision making during math lessons.
Regulation		Stem: When I work with math during lessons, I do it because…
External	ext1	…I want to avoid people nagging at me.
External	ext3	…I do not want the teacher to get angry with me.
Introjected	itj1	...I want the teacher to think I'm a good student.
Introjected	itj2	…I do not want my teacher to get disappointed with me.
Identified	ide1	...it is important to me to get better at mathematics.
Identified	ide2	…it is important to me to learn new things.
Identified	ide3	…it is important to me to understand mathematics.
Intrinsic	int1	…it is fun.
Intrinsic	int2	…I like to work with math.
Intrinsic	int3	…I think math is interesting.
Behavioral Engagement	b2	During math lessons I think about what I should learn all the time.
Behavioral Engagement	b3	I always try to learn as much as possible during the math lessons.
Behavioral Engagement	b4	I use all time during lessons to work with math.
