# Supplementary material for: Relationships Between Basic Psychological Need Satisfaction, Regulations, and Behavioral Engagement in Mathematics
Source: Front Psychol. 2022 Apr 12;13:829958. doi: 10.3389/fpsyg.2022.829958 (PMC9040704; doi:10.3389/fpsyg.2022.829958)
Supplement: Supplementary file 2 [file Table_2.pdf]

**Supplementary Table 2.** Item Loadings on the Latent Variables in the Final SEM-Model

| Item | Latent variable | *β* (*SE*) | *B* (*SE*) |
|---|---|---|---|
| C2 | Competence need satisfaction | 0.812 (0.018) | 0.781 (0.033) |
| C3 | Competence need satisfaction | 0.767 (0.016) | 0.807 (0.029) |
| C4 | Competence need satisfaction | 0.799 (0.016) | 0.809 (0.029) |
| A2 | Autonomy need satisfaction | 0.800 (0.018) | 0.809 (0.031) |
| A3 | Autonomy need satisfaction | 0.721 (0.022) | 0.827 (0.032) |
| A4 | Autonomy need satisfaction | 0.766 (0.019) | 0.839 (0.028) |
| R1 | Relatedness need satisfaction | 0.797 (0.023) | 0.907 (0.039) |
| R2 | Relatedness need satisfaction | 0.878 (0.013) | 1.004 (0.034) |
| R3 | Relatedness need satisfaction | 0.860 (0.014) | 0.996 (0.030) |
| EXT1 | Controlled motivation | 0.574 (0.032) | 0.731 (0.042) |
| EXT3 | Controlled motivation | 0.736 (0.033) | 0.941 (0.047) |
| ITJ1 | Controlled motivation | 0.568 (0.032) | 0.678 (0.043) |
| ITJ2 | Controlled motivation | 0.817 (0.032) | 1.001 (0.049) |
| IDE1 | Identified motivation | 0.831 (0.018) | 0.649 (0.030) |
| IDE2 | Identified motivation | 0.876 (0.014) | 0.727 (0.031) |
| IDE3 | Identified motivation | 0.861 (0.015) | 0.681 (0.032) |
| INT1 | Intrinsic motivation | 0.881 (0.011) | 0.858 (0.024) |
| INT2 | Intrinsic motivation | 0.939 (0.007) | 0.940 (0.026) |
| INT3 | Intrinsic motivation | 0.902 (0.011) | 0.914 (0.024) |
| B2 | Behavioral engagement | 0.771 (0.019) | 0.431 (0.024) |
| B3 | Behavioral engagement | 0.834 (0.017) | 0.432 (0.026) |
| B4 | Behavioral engagement | 0.790 (0.021) | 0.410 (0.028) |

*Note:* *β* = standardized coefficient, *B* = unstandardized coefficient, *SE* = standard error.
