# Supplementary material for: Relationships Between Basic Psychological Need Satisfaction, Regulations, and Behavioral Engagement in Mathematics
Source: Front Psychol. 2022 Apr 12;13:829958. doi: 10.3389/fpsyg.2022.829958 (PMC9040704; doi:10.3389/fpsyg.2022.829958)
Supplement: Supplementary file 3 [file Table_3.pdf]

**Supplementary Table 3.** Standardized Path Coefficients for Latent Variables in the Final SEM-Model

| Relation | Estimate | *SE* | *p* |
|---|---|---|---|
| Engagement on: | | | |
| Controlled regulation | 0.032 | 0.023 | 0.159 |
| Identified regulation | 0.535 | 0.041 | 0.000 |
| Intrinsic regulation | 0.068 | 0.040 | 0.089 |
| Perceived competence | 0.326 | 0.055 | 0.000 |
| Perceived autonomy | 0.042 | 0.041 | 0.307 |
| Perceived relatedness | 0.010 | 0.048 | 0.828 |
| Controlled regulation on: | | | |
| Perceived competence | -0.231 | 0.038 | 0.000 |
| Perceived autonomy | 0.193 | 0.048 | 0.000 |
| Perceived relatedness | -0.054 | 0.047 | 0.249 |
| Identified regulation on: | | | |
| Perceived competence | 0.503 | 0.045 | 0.000 |
| Perceived autonomy | 0.030 | 0.054 | 0.570 |
| Perceived relatedness | 0.167 | 0.060 | 0.006 |
| Intrinsic regulation on: | | | |
| Perceived competence | 0.563 | 0.030 | 0.000 |
| Perceived autonomy | 0.223 | 0.047 | 0.000 |
| Perceived relatedness | -0.051 | 0.044 | 0.239 |

*Note: SE* = standard error
